# Incidence and survival of Lung Cancer: A retrospective population-based cohort (Monastir, Tunisia: 2002–2022)

**DOI:** 10.1371/journal.pone.0338140

**Published:** 2025-12-05

**Authors:** Wafa Dhouib, Amani Maatouk, Cyrine Bennasrallah, Mariem Kacem, Syrine Doghri, Ahmed Trigui, Mounib Bouazizi, Lamis Milad, Manel Ben Fredj, Ines Bouanène, Hela Abroug, Naceur Rouatbi, Sonia Zaied, Moncef Mokni, Asma Belguith Sriha, Imen Zemni

**Affiliations:** 1 Department of Epidemiology and Preventive Medicine, University Hospital Fattouma Bourguiba of Monastir, University of Monastir, Faculty of Medicine of Monastir, Monastir, Tunisia; 2 Technology and Medical Imaging Research Laboratory - LTIM - LR12ES06, University of Monastir, Faculty of Medicine of Monastir, Monastir, Tunisia; 3 National Institut of Public health, University of ElManar, Faculty of Medicine of Tunis, Tunis, Tunisia; 4 Department of Social Medicine, University Hospital of Sfax, Faculty of Medicine of Sfax, Sfax, Tunisia; 5 Department of Family Medicine, University of Monastir, Faculty of Medicine of Monastir, Monastir, Tunisia; 6 Department of community and Preventive Medicine, Regional Hospital of Ksar Hellal, Faculty of Medicine of Monastir, Monastir, Tunisia; 7 Department of pulmonology, University Hospital Fattouma Bourguiba of Monastir, Faculty of medicine of Monastir, Monastir, Tunisia; 8 Department of oncology, University Hospital Fattouma Bourguiba of Monastir, Faculty of medicine of Monastir, Monastir, Tunisia; 9 Cancer Registry of the Center of Tunisia, University Hospital Farhat Hached of Sousse, Sousse, Tunisia; Universiti Monash Malaysia: Monash University Malaysia, MALAYSIA

## Abstract

**Introduction:**

Lung cancer (LC) remains one of the most lethal malignancies worldwide. In Tunisia, LC population-level data is limited. This study aimed to estimate the incidence, mortality, trends burden and survival outcomes of LC over two decades in Monastir (Tunisia).

**Methods:**

We conducted a retrospective population-based cohort including all LC patients diagnosed between 2002 and 2014 in the governorate of Monastir, with follow-up until 2022. Age-standardized incidence and mortality rates (ASIR and ASMR) were calculated and expressed per 100,000 Person-Years (PY). Trends were analyzed using Joinpoint regression to determine Annual Percent Change (APC). Disability-Adjusted Life Years (DALYs), Years of Life Lost (YLL), and Years Lived with Disability (YLD) were computed. Survival analysis was performed using Kaplan–Meier estimates, and multivariable Cox proportional hazards regression was used.

**Results:**

LC ASIR per 100,000 PY was 18.37 (95% CI: 15.1–21.7), 33.63 (29.3–38.0) in males, and 4.50 (2.75–6.41) in females. ASMR per 100,000 PY was 12.33 (9.2–15.84), 24.84 (19.9–29.82) in males, and 2.0 (0.6–3.42) in females. Incidence showed a significant upward trend (APC 8.23%, 95% CI: 2.66–16.12), particularly in females (APC 17.99%, 95% CI: 9.86; 33.27). Projected ASIRs were 43.7 per 100,000 PY in 2025 (95% CI: 37.1–50.3) and 64.7 per 100,000 PY in 2030 (95% CI: 56.7–72.7). DALYs attributable to LC were 1,328 per 100,000 PY, comprising 1,198.8 YLLs and 129.2 YLDs. One-year and five-year survival rates were 43.2% (95% CI: 36.6–49.8) and 22.1% (95% CI: 17.4–26.8), respectively, with a median survival of 10 months (95% CI: 8.9–11.0). Survival was higher among females, younger patients (<65 years), and those diagnosed after 2009, with an improved median survival.

**Conclusion:**

LC incidence and mortality are rising, with low survival and a substantial burden of premature mortality, highlighting the urgent need for population-based prevention programs.

## Introduction

### Background/rationale

Lung cancer (LC) remains a critical global public health challenge, ranking as the most prevalent cancer among men. It stands as the leading cause of preventable cancer-related deaths worldwide [1]. According to the Global Cancer Observatory, an alarming 2,480,675 new LC cases and 1,817,469 related deaths were recorded in the world in 2022 [1]. The alarming rate of premature mortality from LC makes it a pressing global health, social, and economic burden [2,3]. In Tunisia, the estimated Age-Standardized Incidence Rate (ASIR) among males ranges from 25.6 to 38.3 per 100,000 Person-Years (PY) according to GLOBOCAN 2022 [3]. Comparable estimates from neighboring countries indicate slightly lower rates, with Algeria (ASIR 14.8; ASMR 13.7), Morocco (ASIR 31.1; ASMR 28.0), and Egypt (ASIR 17.0; ASMR 15.0) [3]. Many low-income countries have limited data on LC incidence and mortality. In comparison, high-income countries show considerably higher LC incidence and mortality rates: United States (ASIR 86.1; ASMR 51.9), Germany (ASIR 43.2; ASMR 35.5), and the United Kingdom (ASIR 30.1; ASMR 19.4) [3]. These regional and global contrasts underscore the relevance of generating detailed epidemiological data from Tunisia, providing context-specific insights for LC prevention, control strategies, and policy planning in the Maghreb and other low- and middle-income countries.

LC incidence continues to rise, largely fueled by tobacco exposure, the dominant risk factor (RR = 8.96; 95% CI: 6.73–12.11) [4]. Primary prevention remains the cornerstone, focusing on reducing exposure to risk factors. Meanwhile, secondary prevention by the early detection, with low-dose computed tomography (LDCT) proving to identify LC at earlier, more treatable stages [5]. Comprehensive data on the incidence, mortality, and survival of LC are crucial for driving impactful health policies [6]. By analyzing epidemiological trends and LC survival rates, we can allow for more effective interventions [7,8].

### Objectives

This study aimed to estimate the incidence, analyze mortality, trends and survival outcomes, and quantify the burden of LC over two decades in Monastir (Tunisia).

## Methods

### Study design

We conducted a retrospective population-based cohort, tracking all LC patients diagnosed between 2002 and 2014 in the governorate of Monastir (Tunisia), with follow-up until 2022. Our study protocol has been deposited in **protocols.io** to enhance reproducibility (https://doi.org/10.17504/protocols.io.81wgbw4pogpk/v1).

### Setting

Our study was conducted in the governorate of Monastir as a representative Tunisian governorate. The prevalence of smoking among the male population in the region was 50.4% (95% CI [49.49–51.3]) [9]. The prevalence of electronic cigarettes use was 22.7% of cases [10]. The year 2009 was chosen as a turning point because the former President of the Republic officially declared it the ‘National Year for the Fight Against Tobacco.’ Following this declaration, substantial efforts were deployed, including allocation of human and material resources, with the aim of curbing tobacco use as the key determinant of LC incidence and outcomes. Patients with confirmed LC were followed by pulmonologists, oncologists, and radiotherapists, with most cases being diagnosed at a late stage [2,11]. The Cancer Registry of tunisian center was established in 1987 by the late Professor Chadli Bouzakoura. It records all cancer cases from the central region of Tunisia, including the governorate of Monastir. In 1990, the late Professor Mohamed Soltani, head of the Department of Preventive Medicine and Epidemiology, founded the Hospital Morbidity and Mortality Registry, which covers all hospital-based morbidity and mortality cases including all cancer cases within the Monastir governorate. A collaborative effort between the Cancer Registry of center and the Hospital Morbidity Registry was established to ensure comprehensive identification and documentation of all cancer cases in the Monastir governorate. The cancer registry data and the Hospital Morbidity Registry data were collected according to standardized procedures, including active and passive case finding from both public and private healthcare facilities. Routine quality checks and cross-validation with hospital records were conducted to ensure completeness. Private sector cases were included to the extent that they were reported to the cancer registry.

### Participants

Inclusion Criteria: This study encompassed all cases of LC diagnosed in patients residing in the governorate of Monastir between 2002 and 2014, including those diagnosed and treated outside the region. Participant follow-up was ensured through linkage with the Monastir all-cause mortality database, spanning the years 2001–2022.

Exclusion Criteria: Benign and in situ tumors, tumor recurrences, and the metastatic progression were not included in the study.

### Variables

Tumors were classified according to the 10th edition of the International Classification of Diseases (ICD-10), with code C34 (C34.0, C34.1, C34.2, C34.3, C34.8) assigned to malignant neoplasms of the bronchi and lungs. The dataset included key variables: date of birth, sex, year of diagnosis, LC topography and morphology, vital status, and year of death. The minimal de-identified dataset and analysis code have been deposited in a public repository and are publicly accessible. The dataset includes age, sex, year of diagnosis, survival duration/status, and contains no direct identifiers. Dataset and code are available at Zenodo: https://zenodo.org/records/17105325 (https://doi.org/10.5281/zenodo.17105324).

### Data sources/ measurement

Data on LC cases diagnosed between 2002 and 2014 were prospectively collected from the hospital morbidity registry of Monastir and the Center cancer registry. Verification of data conformity was performed by the team of the Department of Epidemiology and Preventive Medicine of Monastir University Hospital. Access to the merged cancer case database was available in late 2019.

In the second step, deaths among the identified cases and their dates were determined using multiple sources: the hospital death database and the Ministry of Social Affairs database, which covers all deaths in Monastir from 2001 to 2022 (data access granted in February 2023 following approval from the National Authority for the Protection of Personal Data). Death certificate data were linked with the the merged cancer case database. To minimize misclassification, with reliability assessed through internal consistency checks and comparison with hospital records. For missing cases, a name-by-name verification was conducted in the municipal death registry, by the Department of Epidemiology and Preventive Medicine of Monastir University Hospital (Appendix). After consolidating all sources, the study coordinator anonymized the database for analysis, available in late 2023.

Age groups were defined as <40, 40–64, and ≥65 years. The study period was divided into two intervals (2002–2008 and 2009–2014), with the latter corresponding to the 2009 National Year for the Fight Against Tobacco.

Missing values were documented in the results. Date of death was unavailable for 22% of cases; such cases were excluded from specific analyses when necessary, with exclusions explicitly reported. No imputation was performed to minimize bias. Histological subtype was available primarily in the cancer registry but largely absent from the morbidity and mortality register; therefore, histology-specific analyses were restricted to cases with documented morphology.

#### Measurements.

The Crude Incidence and Mortality Rates (CIR; CMR) were calculated based on Tunisian National Institute of Statistics data and was expressed per 100,000 PY. Age-Standardized Incidence and Mortality Rates (ASIR; ASMR) per 100,000 PY were calculated using the World Health Organization (WHO) 2013 world standard population. [12]. Age-specific rates were computed for the following age groups: < 40, 40–64, and ≥65 years.

The direct standardization formula was: $ASR=\;\frac{\sum_{i}\left(r_{i}\times w_{i}\right)}{\sum_{i}wi}\times \text{1}\text{0}\text{0},\text{0}\text{0}\text{0}$; r_i_ = age-specific rate in age group i (cases or deaths per person-years); w_i _= population weight for age group i in the standard population. Years of Life Lost (YLL) were calculated as the difference between the standard life expectancy and the age at death. Years Lived with Disability (YLD) were estimated by multiplying the number of incident cases by the duration of disease and by the disease-specific disability weight (DW). For deceased patients, disease duration was defined as the interval between age at diagnosis and age at death, whereas for surviving patients, it was defined as the interval between age at diagnosis and the study endpoint (31 December 2022). Total YLD for each patient was calculated as the sum of the disease duration multiplied by the corresponding DW assigned according to cancer stage:

**Table:** 

Stage	LC duration	LC Stage	Estimated DW
4	Less than 6 months	Terminal	0.906 (95% CI: 0.873–0.932)
3	6 months to 2 years	Metastatic	0.758 (95% CI: 0.710–0.801)
2	2 to 5 years	Early remission	0.738 (95% CI: 0.686–0.785)
1	More than 5 years	Long-term remission	0.600 (95% CI: 0.542–0.656)

Disability-Adjusted Life Years (DALYs) were obtained by summing YLL and YLD over the study period, and DALYs per 100,000 PY were also calculated [13–15].

### Statistical analysis

Data verification and indicator calculations were conducted using Microsoft Excel. Analyses were performed using Joinpoint Version 5.4.0 and IBM SPSS Statistics 21.0. The chi-squared test for independent samples was used to compare incidence and mortality rates between males and females, as well as across different age groups. We used Joinpoint regression analysis to identify significant changes in temporal trends of LC incidence and mortality. This method fits a series of connected linear segments on a logarithmic scale, allowing identification of points (“joinpoints”) where the trend significantly changes. The Annual Percent Change (APC) for each segment is calculated from the slope (*β*) of the regression line as: $APC={(e}^{\beta }-\text{1})*\text{100}$. We used the following formula to project age-standardized incidence rates (ASIR): ${\text{ASIR}}_{t\;}={\text{ASIR}}_{baseline}\times {\left(\text{1}+APC\right)}^{\left(t-baseline\right)}$; Where t is the year of projection [16]). For survival analysis, were included in the merged dataset the patients with a clearly defined vital status. We used the Kaplan–Meier method to estimate overall survival probabilities. Survival curves were plotted for the entire cohort and by age groups, with differences assessed using the log-rank test.

### Ethics approval and consent to participate

We examined anonymized aggregate data. Our study was conducted in accordance to the relevant guidelines and regulations. The Ethics committee of the faculty of medicine of Monastir approved the protocol of this study (IORG 0009738 N°101/OMB0990–0279)

## Results

### Lower respiratory tract cancers (2002-2014)

A total of 1,487 cases of respiratory tract cancers were recorded, corresponding to a CIR of 19.9/ 100,000 PY (95% CI: 15.5–24.4). LC accounted for 86.5% of lower respiratory tract cancers. Laryngeal cancer represented 199 cases (13.4%) with a CIR of 2.67/ 100,000 PY (95% CI: 1.0–4.30), two cases of tracheal cancer were observed.

### Lung cancer incidence (2002–2014)

During 13 years (2002–2014), a total of 1,286 LC cases were observed. The male female ratio was 6.90. The median age at LC diagnosis was 60 years (IQR: 50–70). Men were diagnosed at a significantly older median age of 63 years (IQR: 54–72), compared to 56 years in women (IQR: 46–68) (p < 0.0001).

**Fig 1:**
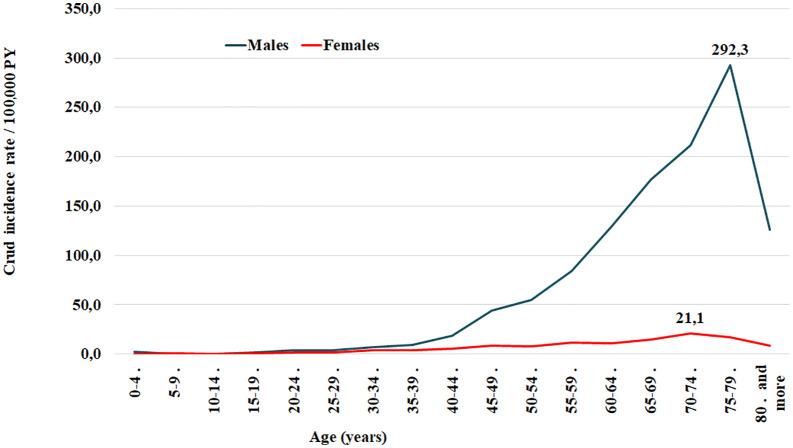
Age-specific incidence of lung cancer by sex, Monastir, 2002–2014. Incidence rates (per 100,000) by age group for men and women. Highest rates observed in men aged 75–79 years.

The CIR was 17.25/100,000 PY. CIR was higher in males (30.24 vs 4.33 in females; p < 0.001). According to sex and age groups, the highest incidence was observed among men aged 75–79 years, reaching 292.3 per 100,000 person-years (Fig 1). ASIR per 100,000 PY was 18.37 (95% CI: 15.1–21.7), 33.63 (29.3–38.0) in males, and 4.50 (2.75–6.41) in females. (Table 1).

**Table 1:** Lung cancer morbidity and mortality: Incidence and trends according to age and sex (2002-2014).

LC Morbidity	All			Males			Females		
	Incidence		Trends	Incidence		Trends	Incidence		Trends
	N (%)	CIR^$^ (95% CI)	APC (95% CI)	N (%)	CIR^$^ (95% CI)	APC (95% CI)	N (%)	CIR^$^ (95% CI)	APC (95% CI)
All	1286 (107/year)	17.25 (13.1;21.4)	8.23 (2.66;16.12)*	1124	30.24 (24.7;35;7)	7.31 (3.17; 12.74)*	163*	4.33 (2.3-6.4)	17.99 (9.86; 33.27)*
Age groups									
< 40	133 (10.6)	5.33 (2.3;8.38)	11.63 (4.09;25.09)*	91 (8.3)	7.44 (3.8; 11.1)	11.03 (4.1; 23.1)*	42 (25.8)	3.28 (1.2; 5.85)	10.35 (−2.7; 25.2)
40-64	640 (51.1)	33.65 (27.9;39.5)	10.19 (5.60;16.66)*	561 (51.5)	58.31 (50.7; 65.9)	9.24 (5.3; 14.6)*	79 (48.5)	8.43 (5.5; 11.34)	18.52 (6.5; 46.4)*
≥ 65	480 (38.3)	95.77 (86.0;105.6)	8.21 (3.28;15.02)*	438 (40.2)	195.23 (181.3; 209.2)	7.24 (3.2; 12.5)*	42 (25.8)	15.23 (11.3; 19.13)	17.43 (6.8; 42.9)*
		**ASIR** ^**$**^ ** (95% CI)**			**ASIR** ^**$**^ ** (95% CI)**			**ASIR** ^**$**^ ** (95% CI)**	
		18.37 (15.1-21.7)			33.63 (29.3-38.0)			4.50 (2.75-6.41)	
LC Mortality	All			Males			Females		
	**N (%)**	**CMR** ^**$**^ ** (95% CI)**		**N (%)**	**CMR** ^**$**^ ** (95% CI)**		**N (%)**	**CMR** ^**$**^ ** (95% CI)**	
All	862 (86.4)	12.53 (8.9;16.0)	8.36 (5.40;12.03)*	791 (89.7)	23.06 (18.3; 27.9)	8.36 (5.39;12.03)*	71 (61.2)*	2.06 (0.6-3.5)	13.81 (3.32; 30.61)*
Age groups at death									
< 40	26 (3.0)	1.05 (0.0; 2,07)	–	18 (2.3)	1.50 (0.2;2.64)		8 (11.3)	0.63 (0; 1.42)	–
40-64	415 (48.8)	21.86 (17.2;26.5)	7.52 (2.52;14.15)*	386 (48.9)	40.12 (33.8;46.46)	7.34 (1.60;14.90)*	29 (40.8)	3.10 (1.3; 4.85)	–
≥ 65	420 (48.8)	83.97 (74.8;93.1)	5.48 (1.13;11.06)*	386 (48.9)	172.05 (158.9;185.2)	4.89 (0.35; 10.65)*	34 (47.9)	12.33 (8.8; 15.84)	11.58 (2.84; 28.44)*
		**ASMR** ^**$**^ ** (95% CI)**			**ASMR** ^**$**^ ** (95% CI)**			**ASMR** ^**$**^ ** (95% CI)**	
		12.33 (9.2;15.84)			24.84 (19.9; 29.82)			2.00 (0.6;3.42)	

CIR/ CMR: Crude Incidence Rate/ Crude Mortality Rate; ASIR/ ASMR: Age-Standardized Incidence Rate/ Age-Standardized Mortality Rate; APC: Annual Percent Change, CI: Confidence Interval; ^**$:**^/100,000 PY.

### Lung cancer mortality (2002–2022)

**Fig 2:**
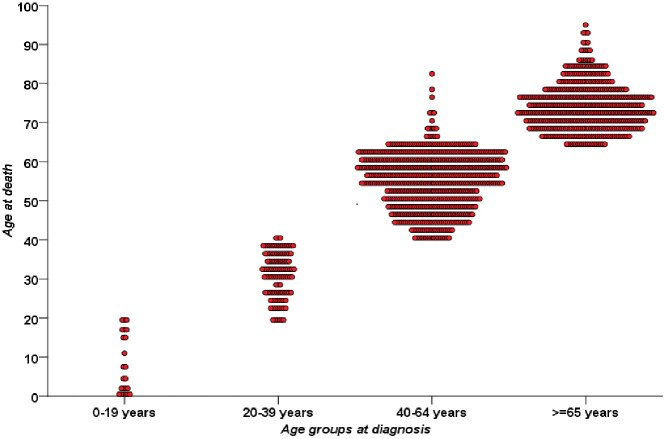
Parallel age distribution of lung cancer incidence and mortality. Lung cancer deaths occurred in the same age group as diagnosis, underscoring limited post-diagnosis survival.

During 20 years of follow up (2022–2022), 862 deaths were documented, CMR was 12.53/100,000 PY. Median age at death was 64 years (IQR: 55–73) equally in males and females. CMR per 100,000 PY was 23.06 in males and 2.06 in females (p < 0.001). Deaths were almost equally distributed between the 40–64 and ≥65 age groups. The highest CMR was observed in individuals aged ≥ 65 years (CMR: 83.97/ 100,000 PY). ASMR per 100,000 PY was 12.33 (95% CI: 9.2;15.84), significantly higher in males (24.84 (95% CI:19.9; 29.82)) than in females (2.00(95% CI: 0.6;3.42)) (Table 1). Deaths occurred predominantly in the same age group as diagnosis, as illustrated in Fig 2.

### Lung Cancer trends and projections

**Fig 3:**
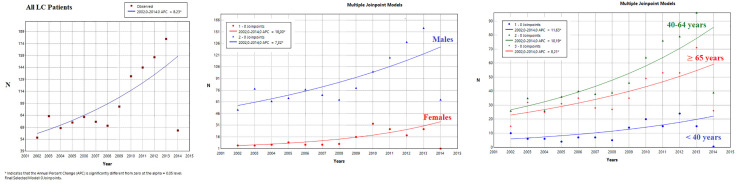
Joinpoint analysis of lung cancer trends (2002–2014) in Monastir (Tunisia). The CIR of LC showed a statistically significant upward trend. This increase was observed in both sexes, and in different age groups.

The CIR of LC showed a statistically significant upward trend, with an APC of 8.23% (95% CI: 2.66;16.12). This rise was higher among females (APC = 17.99% (95% CI: 9.86; 33.27)) compared to males (APC = 7.31% (95% CI: 3.17; 12.74)). Consistent increases were observed across all age groups (Fig 3).

The male female ratio decreased significantly between 2002 and 2009 (APC = –11.46%; 95% CI: –21.3 to –0.3), whereas the median age at diagnosis remained stable over the study period (p = 0.122).

By 2025 the predicted ASIR per 100,000 PY was estimated at 43.7 (95% CI: 37.1–50.31) being 63.5 (95% CI: 55.5–71.5) in males and 22.6 (95% CI: 17.8; 27.35) in females. By 2030 the predicted ASIR per 100,000 PY was estimated at 64.7 (95% CI: 56.7–72.74) being 82.0 (95% CI: 72.9–91.1) in males and 40.9 (95% CI: 34.5; 47.29) in females.

In terms of mortality, the CMR followed a significant upward trend (APC = 8.36%). Among women aged ≥65, the mortality APC reached 11.58%.

### Disability-Adjusted Life Years (DALYs) attributable to lung cancer

The total number of DALYs attributable to LC was 9,901, corresponding to an overall rate of 1,328 DALYs per 100,000 PY. The burden was overwhelmingly driven by YLLs (1,198.8/100,000 PY). YLDs was (129.2/100,000 PY).

By age group, in the 40–64 year age group, the burden accounted for approximately three-quarters of the overall DALYs(3,986 DALYs/100,000 PY), predominated by YLLs(3,751.2). In those ≥65 years, the burden remained substantial (2,089 DALYs/100,000 PY), mortality remained important (1,102.8 YLLs), disability became proportionally more significant (986.1 YLDs). By sex, men carried a disproportionately higher burden, with 2,220.8 DALYs/100,000 PY, largely explained by mortality (2,088.5 YLLs) (Table 2).

**Table 2:** Burden attributable to lung cancer by age group and sex (2002−14; 2022).

	DALY: Totals*	DALY/100,000 PY (95% CI)	YLL/100,000 PY (95% CI)	YLDS/100,000 PY (95% CI)
All	**9901**	**1328.0 (1291.8;1364.2)**	**1198.8 (1164.4;1233.2)**	**129.2 (117.8;140.5)**
< 40	1217.4	240.7 (225.;256.2)	236.0 (220.6;251.3)	4.8 (2.6;7.0)
40-64	7568.4	3986.1 (3924.2;4047.9)	3751.2 (3691.1;3811.3)	234.8 (219.5;250.2)
≥ 65	1044.8	2088.9 (2043.7;2134.1)	1102.8 (1069.8;1135.9)	986.1 (954.8;986.1)
Sex				
Male	8253.8	2220.8 (2174.2;2267.4)	2088.5 (2043.3;2133.7)	132.2 (120.7;143.7)
Female	1192.7	319.0 (301.2;336.8)	295.3 (278.1;312.4)	23.5 (18.7;28.4)

*cohort 2002–2014 followed to 2022; DALYs: Disability-Adjusted Life Years; YLL/ YLD: Years of Life Lost/ Years Lived with Disability; CI: Confidence Interval.

### Lung cancer topography and morphology

The upper lobe (C34.1) was the most frequently affected site (17.1%), with a significant annual increase (APC = 4.10; 95% CI: 1.81–6.79). The lower lobe (C34.3) and overlapping lesions (C34.8) showed significant increases, with APCs of 7.37 and 16.45 respectively. Adenocarcinoma (ADC) was the most common histologic type (41.6%), followed by squamous cell carcinoma (SCC) (29.9%) and small cell lung carcinoma (SCLC) (17.7%). All three subtypes showed significantly increasing trends over time, with adenocarcinoma and small cell lung carcinoma exhibiting the highest APCs (12.10 and 12.33, respectively) (Table 3).

**Table 3:** Lung cancer topography and morphology (2002-2014).

Topography	N (%)	APC (95% CI)
C34.1 – Upper lobe of lung	271 (17.1)	4.10 (1.81;6.79)*
C34.2 – Middle lobe of lung	16 (1.0)	NS
C34.3 – Lower lobe of lung	94 (5.9)	7.37 (1.41;15.94)*
C34.8 – Overlapping lesion of lung	68 (4.3)	16.45 (8.22;36.45)*
C34.0 – Main bronchus	20 (3.1)	;
C34.9 – Lung, unspecified	176 (11.1)	;
Morphology		
Small Cell Lung Carcinoma (SCLC)	114 (17.7)	12.33 (5.20;19.93)*
Squamous Cell Carcinoma (SCC)	193 (29.9)	7.44 (2.30;14.61)*
Adenocarcinoma (ADC)	268 (41.6)	12.10 (7.62;19.14)*
Undifferentiated Carcinoma/ NOS	60 (9.3)	NS
Mixed or rare Carcinomas	10 (1.6)	

*: p < 0.0001; APC: Annual Percent Change.

### Lung cancer survival analysis

**Fig 4:**
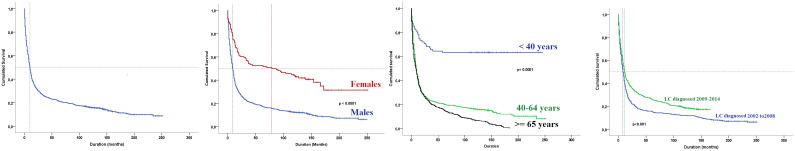
Survival curves of lung cancer by sex and age groups, Monastir, 2002–2022. Kaplan–Meier survival curves by sex and age group. Median survival was 10 months (95% CI: 8.9–11.0), with females showing longer survival than males (79 vs. 9 months).

Out of 1,286 recorded cases, 997 patients (77.5%) were included in the survival analysis (2002–2022). The one-year survival rate was 43.2% (95% CI: 36.6–49.8), the five-years 22.1% (95% CI: 17.4–26.8). The median survival time was 10 months (95% CI: 8.9.1–11.0). Females had a longer survival compared to males (79 vs. 9 months) (Fig 4). Females had a better outcomes in multivariable Cox regression (HR = 0.49, 95% CI: 0.38–0.62, p < 0.0001). Younger patients (<40 years) and the 40–64 years group had a better survival compared to elderly. Survival improved over time, from 8.0 months in 2002–2008 to 11.0 months in 2009–2014 (HR = 0.82, 95% CI: 0.71–0.94, p = 0.004) with a risk reduction of 18%. (Table 4)

**Table 4:** Prognostic factors associated with survival in lung cancer patients (Monastir; Tunisia; 2002–2022).

	Univariate analysis		Cox regression		
	Median survival time: months (95% CI)	p	HR	95% CI HR	p
	10.0 (8.9.1;11.0)				
Males	9.0 (8.0;10.0)	< 0.0001	1		<0.0001
Females	79 (15.7;142.2)		0.491	0.38;0.62	
Age groups					
< 40	–		0.217	0.14;0.32	<0.0001
40-64	9.0 (7.9; 10.0)	< 0.0001	0.813	0.71;0.93	0.003
≥ 65	9.0 (7.0; 10.9)		1		
Morphology					
Small Cell Lung Carcinoma (SCLC)	8 (6.4;9.5)				
Squamous Cell Carcinoma (SCC)	5 (2.9;7.1)	0.407			
Adenocarcinoma (ADC)	6 (3.6;8.3)				
Period					
2002-2008	8.0 (6.6; 9.4)	< 0.0001	1		
2009-2014	11.0 (9.4;12.6)		0.82	0.71;0.94	0.004

CI: Confidence Interval; HR: Hazard Ratio.

## Discussion

### Key results

The ASIR of LC was 18.37 per 100,000 PY (95% CI: 15.1–21.7), with a marked gender disparity. The ASMR was 12.33 per 100,000 PY (9.2–15.84). A significant upward trend in incidence was observed (APC = 8.23%, 95% CI: 2.66–16.12). The projected ASIR was 64.7 (56.7–72.7) per 100,000 PY in 2030. DALYs was1,328 per 100,000 PY, comprising 1,198.8 YLLs and 129.2 YLDs. The one-year and five-year survival rates were 43.2% (95% CI: 36.6–49.8) and 22.1% (95% CI: 17.4–26.8), respectively, with a median survival of 10 months (95% CI: 8.9–11.0). Survival outcomes were significantly better among females, patients under 65 years, and those diagnosed after 2009.

### Interpretation

The elevated ASIR of LC (18.37 per 100,000 PY) is in line with national estimates reported in Tunisia (13–16/100,000 PY in 2012 and 2018) [17,18], and comparable to those observed across North African countries [3].

A marked male female disparity was observed, with a ratio of 6.9, reflecting a substantially higher burden among men. The ASIR in males (33.63/ 100,000 PY) greatly exceeded that in females (4.50/ 100,000 PY). These rates are higher than those reported in 2012, when estimates were 31.1 per 100,000 PY in men and 1.7 per 100,000 PY in women [17]. Similar gender disparities have been documented in other North African countries, where LC incidence closely parallels sex-specific smoking trends [3]. In fact LC incidence is closely linked to smoking patterns, typically 8–15 years earlier [19,20]. In Tunisia, the smoking prevalence in 1996 was 30%, with a pronounced sex difference (55% in men vs. 5.6% in women) [21]. In 2010, over half of men continued to smoke [22]. Given the persistence of high tobacco consumption among men, and the emerging increase in smoking among women and youth, it is likely that the incidence of LC will continue to rise, maintaining the same upward trend observed in this study.

In our study the overall LC incidence increased significantly (APC = +8.23%, *p* < 0.05). Unless effective tobacco control policies and targeted prevention programs are strengthened, the burden of LC may be expected to remain high. This trend coincides with a concerning rise in tobacco use among adolescents and women. Smoking prevalence among adolescents increased from 15.2% in 2013 [23] to 17.3% in 2016 [24], reaching 22.1% in 2022 [25]. Among adolescents in 2013, 26% of boys and 8% of girls were smokers, with the prevalence among girls rising to 11.7% by 2016 [24].

Between 2002 and 2009, the male-female ratio in LC incidence showed a significant downward trend (APC = –11.46), indicating a narrowing male-female gap. Although the female burden remains lower in absolute terms, the relative increase in incidence was notably higher in women (APC = 15.97%) than in men (APC = 5.93%**).** This shift is likely linked to increasing active and passive tobacco exposure among women and younger populations. These findings underscore the urgent need for gender- and age-specific tobacco prevention strategies to mitigate the projected rise in LC incidence in Tunisia.

Approximately half of LC cases occurred among individuals aged 40–65 years, the most economically productive and socially active segment of the population. This age group often represents the main financial support within households, so disease onset in these individuals imposes a substantial societal and economic burden, not only through lost productivity but also through the rising cost of long-term healthcare and palliative management. This burden is further compounded b**y** high nicotine dependence and heavy tobacco use in Tunisia, which continue to hinder cessation efforts [26,27]. The economic impact is therefore expected to rise, as demonstrated by Ju-Fang Shi *et al.* [28].

Predictions indicate that LC ASIR will reache 228.6 per 100,000 by 2030. This steep increase aligns with global trends [29] linked to tobacco use, [30] air pollution, and population ageing. Notably, the rising incidence among women, likely reflecting secondhand smoke exposure, indoor/outdoor pollution, and changing lifestyle patterns [31]. The consistency of our findings with regional and international data, including Globocan, underscores both their plausibility and the urgency of action. These projections highlight the need for strong tobacco control.

The ASMR was 12.33/100,000 PY (95% CI: 9.2–15.84), consistent with previous national reports [17,32]. Mortality trends mirrored incidence, reflecting poor survival due to late diagnosis [33,34]. The insidious clinical presentation of LC, often characterized by non-specific respiratory symptoms, contributes to delayed detection and loss of opportunity for curative treatment [35] Alarmingly, both incidence and mortality increased more sharply in women, echoing global patterns [36]. This contrasts with high-income countries such as the U.S., where LC mortality in women has declined, albeit more slowly than in men (1.1% vs. 2.6% annually) [34]. Given women’s central familial and caregiving roles, their illness generates profound psychosocial and economic consequences [37].

The total number of DALYs attributable to LC was 9,901 (1,328 DALYs/100,000 PY). The burden was overwhelmingly driven by YLLs (1,198.8 per 100,000 PY) confirming the excess mortality and early fatality associated with LC. By age group, individuals aged 40–64 years contributed to nearly three-quarters of the total burden (3,986 DALYs per 100,000 PY), largely explained by premature mortality (3,751.2 YLLs). Among those aged ≥65 years, the burden remained substantial (2,089 DALYs per 100,000 PY), with mortality still predominant (1,102.8 YLLs) but disability gaining proportional importance (986.1 YLDs). By sex, men carried a disproportionately higher burden. These findings, consistent with data from northern Tunisia [38], highlight that LC is a major contributor to excess mortality, particularly among individuals in their most productive years, thereby magnifying its societal and economic impact.

Histological trends revealed adenocarcinoma (ADC) as the most frequent subtype, followed by squamous cell carcinoma (SCC) and small cell lung carcinoma (SCLC), all showing increasing trends. The rise in ADC aligns with global patterns, largely influenced by evolving smoking behaviors and its peripheral lung tropism [38]. In developing countries, the coexistence of various cigarette types including those with higher tar content, poor-quality or low-cost products, and smuggled tobacco may explain to the diverse distribution of LC histological subtypes observed.

The poor survival observed in our cohort (the one-year: 43.2%, the five-years 22.1%) can largely be explained by the very late stage at diagnosis. Many patients tend to underestimate early warning symptoms: chronic cough is often attributed to long-term smoking, while weight loss and anorexia are perceived as direct effects of tobacco rather than signs of cancer. Furthermore, there remains a widespread denial of the causal link between tobacco and LC, which contributes to delays in seeking medical care. At the system level, limited efforts in screening or targeted awareness campaigns for smokers with persistent symptoms further hinder timely detection. Although patients diagnosed with LC do receive the available standard treatments, outcomes remain disappointing due to the predominance of advanced and metastatic disease at presentation. [35,39].

Females had longer survival compared to males. This difference is consistent with international literature, which often attributes the survival advantage in women to biological factors, different tumor histopathology, and possibly lower comorbidity burden [40]. Young adults and adults had a longer survival emphasizing the role of age-related factors such as comorbidities and treatment tolerance [41]. Survival improved over time (2002–2008 vs. 2009–2014) (HR = 0.82). This improvement is likely related to the national tobacco control program launched in 2009. Smoking cessation programs and awareness campaigns have contributed to reducing smoking prevalence and improving the prognosis of patients who successfully quit smoking [42]. These initiatives may also have encouraged smokers for earlier medical consultation and diagnosis. Alternatively, the observed improvement could reflect the introduction of new lung cancer treatments following the 2009 budget allocation for tobacco control and cancer management.

## Conclusion

LC remains a major public health challenge, with rising incidence and persistently high mortality, particularly among men, though an alarming upward trend is observed among women. Survival outcomes remain poor, reflecting late-stage diagnosis and limited access to advanced therapies. The increasing burden, expressed through high DALYs, underscores the urgent need for strengthened tobacco control, early detection strategies, and improved access to diagnostic and therapeutic services. These findings highlight the importance of sustained national cancer surveillance and targeted prevention policies to mitigate the growing LC impact.

### Strengths and limitations

This population-based study offers a comprehensive analysis of LC burden in the Monastir governorate over a 20-year period (2002–2022). Leveraging complete incidence data (2002–2014) with vital status follow-up through 2022, the study ensures strong external validity. The longitudinal design enabled robust trend analyses for incidence, mortality, and survival, using internationally standardized indicators (CIR, CMR, ASIR, ASMR, APC), enhancing comparability across studies. Forecasts to 2025 and 2030 provide valuable support for health system planning. Survival analysis adds key insights into prognosis in this high-mortality cancer.

Despite its strengths, the study has some limitations. First, survival analysis excluded 22.5% of cases due to missing data, which may have introduced selection bias and potentially underestimated survival rates. Specifically, patients with missing vital status or date of death were not recorded in the mortality database, and their status could not be confirmed via cross-checks with cancer database. To maintain analytical rigor, only cases with confirmed vital status were included in the survival analysis. Importantly, these excluded patients were retained in the incidence analysis to ensure accurate estimation of crude and age-standardized incidence rates (CIR and ASIR). Despite these exclusions, the survival analysis remains robust, as the overall sample size was sufficient, including for subgroup analyses. Second, the absence of tumor stage information and the lack of individual risk factor data including smoking history, occupational exposures, and comorbidities, limit the interpretation of survival outcomes and preclude causal inference. This limitation reflects the structure of the hospital morbidity and mortality registry during the study period, which was paper-based. To ensure data completeness, the number of collected variables was necessarily restricted, excluding TNM classification for cancers and detailed microbiological or virological characteristics for infectious diseases. Nevertheless, several local studies, including theses, have shown that LCs were predominantly diagnosed at very advanced stages, with the involvement of pulmonologists and oncologists largely confined to palliative care.

Finally, although this study is region-specific (Monastir), the comprehensiveness of the data, the efforts made to capture mortality information for the entire cohort, and the robustness of these data provide a reliable estimate that likely reflects trends in the majority of Tunisian regions and in other developing countries.

## Abbreviations

LC: Lung Cancer
ASIR/ ASMR: Age-Standardized Incidence Rate/ Age-Standardized Mortality Rate
CIR/ CMR: Crude Incidence Rate/ Crude Mortality Rate
APC: Annual Percent Change
DALYs: Disability-Adjusted Life Years
YLL/ YLD: Years of Life Lost/ Years Lived with Disability
95% CI: 95% Confidence Interval
IQR: Interquartile Range
ADC: Adenocarcinoma
SCC: Squamous Cell Carcinoma
SCLC: Small Cell Lung Carcinoma
HR: Hazard Ratio.
